# Phytopharmacovigilance in the Elderly: Highlights from the Brazilian Amazon

**DOI:** 10.1155/2019/9391802

**Published:** 2019-02-03

**Authors:** Carolina Miranda de Sousa Lima, Mayara Amoras Teles Fujishima, Bráulio Érison França dos Santos, Bruno de Paula Lima, Patrícia Carvalho Mastroianni, Francisco Fábio Oliveira de Sousa, Jocivânia Oliveira da Silva

**Affiliations:** ^1^Toxicology Laboratory, Pharmacy Course, Departament of Biological and Health Sciences, Federal University of Amapá, Juscelino Kubitschek Highway, KM-02, Jardim Marco Zero, ZIP Code: 68.903-419, Macapá - AP, Brazil; ^2^Medicine Course, Department of Biological and Health Sciences, Federal University of Amapá, Juscelino Kubitschek Highway, KM-02, Jardim Marco Zero, ZIP Code: 68.903-419, Macapá - AP, Brazil; ^3^Faculty of Pharmaceutical Sciences, State University Paulista Julio Mesquita Filho, Araraquara, Rodovia Araraquara-Jaú KM 01, Machados, ZIP Code: 14800-901, SP, Brazil; ^4^Quality Control and Bromatology Laboratory, Pharmacy Course, Department of Biological and Health Sciences, Federal University of Amapá, Juscelino Kubitschek Highway, KM-02, Jardim Marco Zero, ZIP Code: 68.903-419, Macapá - AP, Brazil

## Abstract

Practices described as traditional medicine may coexist with formalized, science-based medicine. In this context, the present study aimed to verify the profile of the elderly who consumed herbal medicines concomitantly with medications and to identify suspected adverse drug reactions (ADRs) in the Brazilian Amazon (Macapá, Amapá). The study was carried out in two steps: a cross-sectional study (structured questionnaire) and a clinical study (pharmacotherapeutic follow-up). Out of 208 participants, 78.8% were female with age between 60 and 69 years (58.7%), 59.1% used herbal medicines concurrently with medications, and 40.9% did not report use of herbal medicine. Losartan was the most used medication, and* Lippia alba* (Mill.) N.E. Br was the most common herbal medicine used. The total prevalence of suspected ADRs, among the elderly who answered the structured questionnaire, was 41.3%, with 27.4% being in the elderly who used herbal medicines and medications, and 13.9% being in the elderly who used only medications. Meanwhile, the total prevalence of suspected ADRs was 71.0% among the elderly patients who underwent pharmacotherapeutic follow-up, 60.5% in elderly who used herbal medicines and medications, and 10.5% in elderly who used only medications. The most reported ADR symptoms were related to disorders that affect the nervous system (38.4%) in the structured questionnaire and related to digestive disorders (36.4%) in the pharmacotherapeutic follow-up. The probability associated with the occurrence of a given ADR in the face of a set of demographic, socioeconomic, and clinical variables was estimated; the results showed that, in the studied population, only sex (p = 0.030) had an influence on the occurrence of ADR. The prevalence of ADRs with probable causality was high in this study population, but it was only sex-related, although more prevalent in the elderly who consume herbal medicines.

## 1. Introduction

Herbal medicines are widely used in healthcare worldwide, mainly in local communities that have a long history of their use in traditional medicine, defined by World Health Organization (WHO) as “the sum total of the knowledge, skills, and practices based on the theories, beliefs, and experiences indigenous to different cultures, whether explicable or not, used in the maintenance of health as well as in the prevention, diagnosis, improvement or treatment of physical and mental illness” [1]. In Brazil, traditional medicine was historically built from a combination of knowledge and practices of different peoples, especially indigenous groups, Europeans, and Africans [2, 3].

The phytomedicine (the use of herbal medicines with therapeutic properties) in the Brazilian Amazon has emerged from a long historical tradition of using products from nature for curing diseases. Several factors contribute to the increased demand for these products, such as rich biodiversity, cultural knowledge, and social and economic factors [4]. Medicinal plants are customarily cultivated or extracted from native vegetation and are increasingly being purchased in local markets, pharmacies, and other establishments.

Many countries have practices described as traditional medicine which may coexist with formalized, science-based, and institutionalized systems of medical practice represented by biomedicine, here defined as the hegemonic medical system based on the principles of Western science, where both are considered as complementary [4–6].

Since the late 70s, in various statements and resolutions, the WHO has expressed its commitment to encourage the formulation and implementation of public policies for integrated and rational use of traditional medicine (complementary/alternative medicine) and biomedicine in national healthcare, as well as the development of studies for better scientific knowledge about its safety and efficacy [7]. The documents “WHO Strategy on Traditional Medicine 2002–2005” [8], “WHO Guidelines on Safety Monitoring of Herbal Medicines in Pharmacovigilance Systems” [9], and “National Policy on Traditional Medicine and Regulation of Herbal Medicines” [10] reaffirm the development of these principles.

In 2006, Brazil's Ministry of Health Brazilian established a National Policy for Integrative and Complementary Practices (PNPIC), which include traditional medicine. This policy caters mainly the need to understand, support, incorporate, and implement experiences with integrative practices (which include traditional medicine) that had already been developed in primary healthcare in many cities and states [11, 12]. This system is contemplating the doctrinal principles of Unified Health System (SUS) as universality, equity, and integrality and helps to strengthen the system, which is a social victory of the Brazilian people [7].

During the last years, many countries have established or initiated the process of establishing national regulations regarding herbal medicines which is a key mean to ensure the safety, efficacy, and quality of herbal medicinal products. Adverse events arising from consumption of herbal medicines may be due to any one of a number of factors. These include the use of the wrong species of plant by mistake, adulteration of herbal products with other, undeclared medicines, contamination with toxic or hazardous substances, overdosage, misuse of herbal medicines either by the healthcare providers or the consumers, and use of the herbal medicines concomitantly with other medications. Therefore, the analysis of adverse events related to the use of herbal medicines is more complicated than in the case of the medication [9, 10].

Ethnobotanical/ethnopharmacological studies have been used extensively to describe uses, doses, dosages, and sources and methods of preparation of traditional herbal medicines, but their application to date in examining adverse effects, responses to adverse effects, contraindications, toxicity, and other aspects relevant to safety is limited [13].

In recent years, there has been increasing recognition of the need to develop pharmacovigilance (safety monitoring) systems for herbal medicines. In Brazil, as in other countries, medicinal herbs are traditionally considered to be “natural and therefore free of risks” [13, 14]. Pharmacovigilance practices and tools though have developed in the context of the biomedicine, have rarely considered the complexities of monitoring the safety of medicines sourced from plants [15], and require collecting more information about their methods of preparation, administration, adverse events, contraindications, and precautions [13].

Herbal medicines use is relatively common among elderly. However, these individuals are considered a ‘special population' because they differ from younger adults in terms of comorbidity, polypharmacy, pharmacokinetics, and greater vulnerability to adverse drug reactions (ADRs) [16–18] defined as any harmful, unintentional, or undesirable effect caused by a medication at doses used in humans for prophylaxis, diagnosis, or therapy [19–21]. Therefore, an herbal medicine surveillance scheme is essential to promote their safe use among the elderly and identify probable ADRs.

In this context, the present study aimed to verify the profile of the elderly who consumed herbal medicines concomitantly with medications and to identify suspected ADRs through a structured questionnaire and pharmacotherapeutic follow-up in the Brazilian Amazon (Macapá, Amapá).

## 2. Methods

**Study design and setting**. This study was carried out in two stages: (1) a cross-sectional study and (2) a clinical study used to obtain further information, especially on suspected ADRs. All steps were carried out from May 1, 2016, to October 1, 2017, at the Frei Daniel de Samarate Primary Healthcare Unit in the city of Macapá (latitude 00°02′18.84′′N and longitude 51°03′59.10′′W), located in the north of Brazil. The town has an estimated area of 6,503.458 km^2^, with a population of over 398,204, out of which 20,508 are elderly individuals [27]. Macapá is situated in the Plateau of the Guianas in the southeast of the state of Amapá, and the state is bounded by the state of Pará in the west and south; by French Guiana in the north; by the Atlantic Ocean to the northeast; by the mouth of the Amazon River to the east; and by Suriname to the northwest, with few land connections with other parts of Brazil [27].

**Participants (recruitment, inclusion, and exclusion criteria).** Elderly users of the basic health unit who met the following inclusion criteria were invited to participate in the study: people were at least 60 years of age, were nonindigenous (according to ethical criteria (because studies involving indigenous people and their knowledge/culture must follow specific ethical recommendations), were in perfect mental health (determined through medical record review), and have had provided free and informed consent. Those who did not meet the inclusion criteria were excluded from the study.

**Variables**. Data collection was performed using structured questionnaires and pharmacotherapeutic follow-up. The information obtained included participants sociodemographic characteristics (age, sex, marital status, income, schooling, and income source), clinical factors (pharmacotherapy, polypharmacy: ≥5 drugs [23], herbal medicines used, pharmacotherapeutic experience, the results of laboratory tests, therapy safety, social drug use, immunizations, allergies, and alerts) and suspected ADRs.

**Data sources/measurement**. In the structured questionnaire, prepared by the authors, data were obtained through face-to-face interviews, and pharmacotherapy analysis included prescription medications, over-the-counter (OTC) medications, and herbal medicines. The instrument used for the research (structured questionnaire) is a method used widely in collecting pharmacoepidemiological data [28, 29]. We considered as medicines over-the-counter (OTC) those reported by the elderly to be used without guidance/medical prescription and which were contained in the OTC list [30], which defines medicines that can be sold without a prescription in Brazilian territory, and they were analyzed as the other medicinal products, without distinction.

Pharmacotherapeutic follow-up is a practice that can be performed by several methods—such as SOAP, Subjective Objective Assessment Plan [31]; Dader [32]; and the PWDT, Pharmacist's Workup of Drug Therapy [33, 34]—and was developed by pharmacists in response to a need for ongoing treatment of medication-based health problems and to help achieve the patient's therapy goals, thereby optimizing the patient's medical experience. The pharmacotherapeutic follow-up is very useful and efficient for the detection of drug-related problems (DRP) that may indicate suspected adverse drug reactions.

In this study, the method used was the Pharmacist's Workup of Drug Therapy (PWDT) [33, 34], the standard for pharmacotherapy follow-up and ADR investigation. The plan of pharmaceutical care was built up in the first consultations, according to the recommendations of the chosen method, starting from the detection of drug-related problems (DRP) and analysis of these problems, to define the necessary interventions. Subsequently, the impact of the interventions was assessed through their clinical significance and codes that describe whether the intervention was appropriate, indifferent, or inappropriate [35, 36]. The entire working procedure during the consultations was duly documented and recorded as recommended by the method [33, 34] and this information was also used to observe or measure the patients positive experience with drug therapies (effectiveness) and to verify or measure any undesirable effects the patient may have experienced during the drug therapy (safety). Only the initial steps of the follow-up (drug-related problems and analysis of these problems) were analyzed in the present study and no information was used on the interventions.

From both instruments, it was necessary to obtain information regarding (1) the identification of suspected ADR related to herbal medicines and medications and (2) identification of the drug therapy problems, especially those concerning safety. The observations and inferences were analyzed in pairs. Confirmation and management of the suspected ADRs were carried out by evaluating the potential causality and temporal association between the occurrence of the event and the use of medications [16–20] or/and comparing the events in our study with ADRs previously reported in the scientific literature.

**Bias.** Information about the possible ADRs was initially obtained through a structured questionnaire; suspected cases of ADR were then sent to the pharmacotherapeutic follow-up service for a more detailed evaluation. However, adherence to the service was low and may have led to an underestimation of the information.

**Sample size and quantitative variables**. All the elderly who met the inclusion criteria were enrolled in the study, totaling 208 participants. The selected patients answered the structured questionnaire, and after analysis of the data, those with suspected ADR were invited to participate in the next step, the pharmacotherapeutic follow-up; of those invited, only 38 agreed to participate.

**Statistical analyses**. BioEstat® 5.3 software was used for statistical analyses; the hypotheses were bidirectional (*μ*1 ≠ *μ*0), and *α* = 0.05. Descriptive statistic (mean, standard deviation, frequency) was used to characterize the population and its variables. Student T-test was also used to check the difference between medication used, health problems, polypharmacy, and ADR potentially (discrete quantitative variables). Logistic regression was used to estimate the probability associated with the occurrence of a given event (ADRs) in the face of a set of explanatory variables (demographic, socioeconomic, clinical variables).

**Ethical aspects.** This study was performed following the Code of Ethics of the World Medical Association. It was approved by the Human Research Ethics Committee of the Federal University of Amapá (CAAE 38400314.9.0000.0003), and all the patients signed a free and informed consent term authorizing the study.

## 3. Results

In total, 208 patients were interviewed, representing 1% of the elderly population of the city of Macapá [9] and 1.2% of the elderly population assisted by the Brazilian Health Unit System.  Table 1 shows that the mean age of the elderly participants was 69.4±7.5, and the majority of the participants in the study were female (79.8%); 60 to 69 years old, the youngest age group (58.7%); either not married, widowed, or divorced (68.8%); and educated at the primary level (51.1%). Additionally, most study participants had an average income of ≤$588.80 (74.0%).

Out of the 100% patients (208) analyzed, 59.1% (123) used herbal medicines concurrently with medications and 40.9% (85) did not report the use of any herbal medicine in their pharmacotherapy. Hypertension, rheumatic diseases, diabetes, gastritis, and dyslipidemia were the most prevalent diseases (Table 2), constituting the average number of diseases with a value of 2.2±1.1, and elderly people who used herbal medicines in combination with medications presented more health issues (1.6±1.0) compared with patients who used only medications (1.9±1.0). Most of the elderly (81.7%) did not practice polypharmacy (≥5 medications).

In order to estimate the probability associated with the occurrence of a given event (ADRs) in the face of a set of demographic, socioeconomic, and clinical variables, a multiple logistic regression was performed. The dependent variable (Y) was the suspected ADR, and the independent variables were age, schooling, sex, number of health problems, polypharmacy, and usage of herbal medicines. The results showed that in the studied population, only sex (p = 0.030; CI 95% 0.23 to 0.93) had an influence on the occurrence of suspected ADR. However, when estimating the Y value, it was possible to observe that the elderly who use herbal medicines have a 93.4% probability of developing ADR, while the elderly who do not use herbal medicines have a probability of 90.51%.

The medications most commonly prescribed (structured questionnaire) and used were losartan, glibenclamide, omeprazole, and metformin (Table 3), and the mean number was 2.9±1.4 by patient.

Table 4 shows the most frequently reported herbal medicines used by elderly participants, according the structured questionnaire, along with their botanical names, reported properties, and uses.* Lippia alba *(Mill.) N.E. Br (Cidreira, 19.9%) and the* Peumus boldus *Molina (Boldo, 11.1%) were the most frequently consumed.

According to the elderly, the herbal medicines were mostly obtained in fairs or popular markets (51.6%) and in garden (37.0%), while health establishments and pharmacies were the last options (11.4%). Presentations of herbal medicines especially used were infusion/tea (59.5%) and plant extracts (27.5%). Oral use (84.2%) was the most common mode of use of herbal medicines in this population (Table 5).

Compared with the results obtained in the structured questionnaire, the pharmacotherapeutic follow-up was performed through the PWDT methodology, standard method, and validated for clinical follow-up of pharmacotherapy. All the elderly with potential ADRs were invited to participate in this stage of the study, but only 38 accepted, 33 of whom were elderly who used herbal medicines and medicines, and 5 used only medicines. The medicines most commonly prescribed and used by the elderly who underwent pharmacotherapeutic follow-up were losartan and omeprazole (Table 6).

Table 7 shows the most frequently reported herbal medicines used by the elderly participants of pharmacotherapeutic follow-up, along with their botanical names, reported properties and uses. The* Peumus boldus *Molina (Boldo, 19.4%) and* Lippia alba *(Mill.) N.E. Br (Cidreira, 16.7%) were the most frequently consumed, as well as the refueling in the pharmacotherapeutic follow-up.

Regarding the potential ADRs, among the elderly who answered the structured questionnaire, there was a total prevalence of 41.3%, with 27.4% being in the elderly who used herbal medicines and medicines, and 13.9% in the elderly who used only medicines. Among the elderly people with suspected ADRs selected by the structured questionnaire who agreed to continue the investigation, 71.0% (27) had their ADRs confirmed. It was only possible to define the ADRs in the structured questionnaire and pharmacotherapeutic follow-up as shown in Table 8.

The most frequently reported ADR symptoms were related to nervous system disorders (38.4%) in the structured questionnaire and related to digestive disorders (36.4%) in the pharmacotherapeutic follow-up (Table 9).

## 4. Discussion

As a result, increased use of herbal medicines in the Brazilian primary healthcare system has been stimulated [11–37] not only because of the international trend toward the use of more natural treatments but because these treatments are part of the local culture. Therefore, facilitating improved communication in pharmacovigilance is necessary [38, 39] by creating databases for phytotherapy programs and developing and implementing better methods for causal investigation of adverse reactions to herbal medicines.

It was possible to associate suspected ADR with sex, indicating that women are more likely to develop ADR, as already shown in other studies where hormonal factors may influence the establishment of an ADR [40, 41]. Besides that, within the elderly population in this study, we observed a high prevalence of the use of herbal medicines, as the majority of the participants were females, which may have influenced the results. The high consumption of herbal medicines associated with the high level of female participation in this study is supported by the findings of gender-based comparative studies of the knowledge about medicinal plants. Regarding social roles, women are classified as wives and daughters who oversee family health, including diagnosing illnesses and knowing their prognosis; they are also responsible for implementing the first treatments [42–44].

Most of the elderly participants in this study were 60 to 69 years of age, the youngest category, probably due to the demographic characteristics of the region, where the life expectancy is not high. Age did not show a significant influence on the occurrence of ADRs, although many studies indicate an increased risk of ADRs with age [15–41], so studies in this population with a larger age group should clarify this probability better.

Polypharmacy is an important concern for elderly people because they use multiple medications for long periods of time, increasing the likelihood of medication interactions and ADRs [45–47]. The clinical profile of the elderly in this study was relatively comparable to their pharmacotherapeutic profile; specifically, the most prevalent diseases were hypertension, rheumatic diseases, diabetes, and gastritis, and the medications used to treat them were losartan, glibenclamide, and omeprazole. These data also demonstrated that rheumatic diseases, although reported by the participants, were not frequently treated using medications.

While medications are primarily used for blood pressure problems, general pain, and endocrine and nutritional diseases [4], herbal medicines typically are used to treat simple conditions such as digestive and respiratory problems and general pain [48]. This is supported by the data in the present study, wherein the herbal medicines most often reported by the elderly participants were* Lippia alba* (Mill.) N.E. Br and* P. boldus* (Molina); the main indications for both of these medicines are for relaxation and digestive problems, and digestive system problems were the third most cited health problem.

Studies of the medicinal use of herbs in Brazil have shown that the most used dosage forms were infusion and decoction, followed by the use of fresh herbs and their use in bathing [49]. In this study, the most frequently reported pharmaceutical formulations were infusion/tea with herbs. Most likely, infusion/tea is most commonly used due to the simplicity of the preparation techniques. Findings from other studies corroborated this, showing that the main sources of herbal medicines were free markets, traditional healing homes, other sources, and lastly drugstore [49, 50]. The methods of administration of the herbal medicines identified in this study were oral and topical, but another study demonstrated that in African populations, the main routes of administration, in addition to oral and topical, also included respiratory [51].

It is important to note that certain ethnobotanical/ethnopharmacology aspects can be influenced by the regional, environmental, conservation, and storage factors of herbal medicines. For the pharmacovigilance of herbal medicines, the composition of the medicine, the therapeutic use, the preparation and storage, the route of administration, the dose, and the duration of administration are important factors. Concerns about special patient groups, including children and older patients, emphasize the importance of collecting this information in pharmacoepidemiological studies of medicinal plants [13]. In addition to providing more detailed information on the standards for use, new tools for investigating the causality of ADRs associated with herbal medicines [52] have been developed to better elucidate suspected cases.

Although the pharmacotherapeutic follow-up (PWDT) is a recommended method to assess the safety of pharmacotherapy [33, 34], it is not readily applicable in places where there is a scarcity of pharmacists or inadequate infrastructure and training. Besides, the population does not recognize yet the benefits and necessity of pharmacotherapeutic monitoring, demonstrated in this study with the lack of availability by the elderly population to be monitored. Therefore, it is possible to suggest the necessity and feasibility of using the structured questionnaire as a screening tool for ADRs that may help establish an active phytopharmacovigilance in regions without pharmacotherapeutic follow-up services widely available and without the infrastructure for its implementation.

It is important to emphasize that a suspected ADR needs to be evaluated through algorithms to determine the causality of an ADR as described by Naranjo [53], Karch & Lasagna [54], WHO [20], and Mastroianni et al. [52]. This demonstrates how important it is to evaluate pharmacotherapy and the complexity of investigating ADRs associated with herbal medicines. It was also observed that the identification of definitive ADRs was possible only through pharmacotherapeutic follow-up, but probable and possible events were identified by both tools (structured questionnaire and pharmacotherapeutic follow-up). ADRs were very frequently identified using the questionnaire, probably because, unlike the pharmacotherapeutic follow-up, only limited information is needed.

It was also possible to verify that polymedication may increase the probability of ADRs because the average number of medications identified by elderly participants in the pharmacotherapeutic follow-up was much higher than the average number of medications reported in the structured questionnaire, corroborating other studies [19–55]. The classification of ADRs according to the WHO system [20] revealed the high frequency of ADRs related to the nervous and digestive systems, suggesting the hypothesis that herbal medicines are being used to treat ADR symptoms because they are used as a relaxation and in the combat of digestive discomfort or pain and not health problems, as described by the elderly and the classifications of ADRs. Another explanation is that herbal medicines are generally used to treat simple diseases such as digestive, respiratory, or general pain [4]

Encouraging routine reporting of adverse events related to herbal medicines and promoting studies of the interaction between herbal medicines and medications are also essential so that this information can be used to guide clinical practice. In addition, to be able to effectively recommend the use of phytotherapy as a therapeutic option for health system patients, increased investment in studies to develop more reliable data collection methods according to the existing recommendations [56] is necessary to obtain better information for both passive and active pharmacovigilance. Information obtained from spontaneous reports, case series, cross-sectional studies, case-control studies, and cohort studies is important [19–21, 23, 27–29] to better evaluate the risks and consequences of the use of herbs in combination with medications. As a result, more data regarding the safety and efficacy of phytotherapy would be generated, leading to a greater incentive for biomedical medicine to provide more feasible integrative medicine services.

Limitations of the study are as follows: botanical identification of medicinal plants has not been done; some variations in the scientific species may occur; in addition, the sample size of the study can be also considered as one of the limitations.

## 5. Conclusion

This study showed that in a region of the Brazilian Amazon (Macapá, Amapá), the elderly people who consume the most herbal medicines are younger, female, of low-income, and literate. The prevalence of ADRs with probable causality was high in this study population, but it was only sex-related, although more prevalent in the elderly who consume herbal medicines.

Regarding the potential ADRs, among the elderly who answered the structured questionnaire, there was a total prevalence of 41.3% of ADRs, with 27.4% being in the elderly who used herbal medicines and medicines, and 13.9% being in the elderly who used only medicines. it was also possible to observe that when used, the herbal medicines had as main objective to combat symptoms of diseases or, possibly, to combat ADR symptoms caused by the medications used to treat chronic diseases. The results of this study showed the need to actively investigate suspected ADRs, and the structured questionnaire used was an effective and low-cost alternative tool for the screening of suspected ADRs in this study population. In view of the unique regional characteristics, adequate phytopharmacovigilance systems with multiple approaches are needed to overcome the special challenges, and the structured questionnaires as well as a therapeutic follow-up can be useful approaches to increase the likelihood of ADR detection

## Figures and Tables

**Table 1:** Demographic and socioeconomic characteristics of the elderly participants obtained through the structured questionnaire (N = 208), Macapá, Brazil, 2016-2017.

**Demographic and Socioeconomic Index**	**Structured**	
	** questionnaire**	
	**N**	%
**Gender**		
Female	166	79.8
Male	42	20.2
Total	208	100
**Age group (years)**		
60-69	122	58.7
70-79	62	29.8
≥80	24	11.5
Total	208	100
**Marital status**		
Not married, widower and divorced	143	68.8
Married	65	31.2
Total	208	100
**Education level **		
Not formal education	48	23.1
Primary education	105	50.1
Secondary or postsecondary education	55	26.4
Total	208	100
Household income/ **month**^*∗*^		
≤$588.80	154	74.0
>$588.80 ≥$2.650.00	46	22.1
>$2.650.00	08	3.9
Total	208	100

^*∗*^In US dollars according to the Brazilian Central Bank [22] in 01/08/2018 (R$3.24).

**Table 2:** Clinic characteristics regarding only medication and herbal medicines in combination with medication use reported by the elderly participants (N=208), Macapá, Brazil, 2016-2017.

**Clinic Index**	**Only ** **medications use n (**%**)**	**Herbal medicines and medications use n (**%**)**	**Total ** **n (**%**)**	**p value** **∗** **∗**
Health problems				p = 0.004
Hypertension	58 (35.8)	64 (33.0)	122 (34.3)	
Rheumatic diseases	29 (17.9)	44 (22.7)	73 (20.5)	
Diabetes	19 (11.7)	28 (14.4)	47 (13.2)	
Heart problems	12 (7.4)	8 (4.1)	20 (5.6)	
Gastritis	4 (2.5)	12 (6.2)	16 (4.5)	
Dyslipidemias	8 (5.0)	6 (3.1)	14 (3.9)	
Depression	4 (2.5)	4 (2.1)	8 (2.25)	
Labyrinthitis	2 (1.2)	6 (3.1)	8 (2.25)	
Others	26 (17.2)	22 (11.3)	48 (13.5)	
Total	162 (100)	194 (100)	356 (100)	
Polypharmacy*∗*				p < 0.0001
Yes	10 (11.8)	36 (29.3)	46 (22.1)	
No	75 (88.2)	87 (70.7)	162 (77.9)	
Total	85 (100)	123 (100)	208 (100)	
Adverse Drug Reaction (ADR) suspected				
Yes	29 (34.1)	57 (46.3)	86 (41.3)	p = 0.045
No	56 (65.9)	66 (53.7)	122 (58.7)	
Total	85 (100)	123 (100)	208 (100)	

^*∗*^Classification according to Kennerfalk et al. (2002) [23]. Polypharmacy: ≥5 medicines.

*∗∗*Student T-test.

**Table 3:** Medications reported by the elderly participants on the structured questionnaire (N=208), Macapá, Brazil, 2016-2017.

**Medications**	**ATC** **∗**	**Only medication use** **n (**%**)**	**Herbal medicines and medication use n (**%**)**
Acetylsalicylic acid	N02BA01	15 (6.0)	10 (2.8)
Alprazolam	N05BA12	3 (1.2)	0 (0.0)
Amiodarone	C01B	1(0.4)	7 (2.0)
Amitriptyline	N06AA	1(0.4)	1 (0.3)
Amlodipine	C08CA01	2 (0.8)	6 (1.7)
Atenolol	C07A	2 (0.8)	4 (1.1)
Atenolol	C07AB03	5 (2.0)	4 (1.1)
Calcium	A12A	8 (3.2)	10 (2.8)
Captopril	C09AA01	6 (2.4)	7 (2.0)
Carisoprodol	M03	6 (2.4)	10 (2.8)
Carvedilol	C07A	2 (0.8)	4 (1.1)
Chlorpheniramine	R06AB02	2 (0.8)	3 (0.8)
Clopidogrel	B01A	2(0.8)	3 (0.8)
Compounded drugs		11 (4.3)	15 (4.2)
Diazepam	N05BA01	5 (2.0)	5 (1.4)
Diclofenac	M01AB05	8 (3.2)	12 (3.4)
Digoxin	C01A	1(0.4)	3 (0.8)
Dimenhydrinate	A04AD	3 (1.2)	4 (1.1)
Esomeprazole	A02B	1(0.4)	1 (0.3)
Ferrous Sulphate	B03A	2 (0.8)	1 (0.3)
Glibenclamide	A10BB01	10 (4.0)	19 (5.3)
Haloperidol	N05B	1(0.4)	1 (0.3)
Hydrochlorothiazide	C03AA03	9 (3.6)	7 (2.0)
Ibuprofen	M01A	5 (2.0)	12 (3.4)
Insulin	A10AC01	3 (1.2)	8 (2.2)
Losartan	C09AA01	26 (10.3)	33 (9.2)
Meloxicam	M01AC06	6 (2.4)	1 (0.3)
Metformin	A10BA02	6 (2.4)	13 (3.6)
Naproxen	M01A	3 (1.2)	4 (1.1)
Nifedipine	C08CA05	5 (2.0)	6 (1.7)
Nimesulide	M01AX17	1 (0.4)	9 (2.5)
Omeprazole	A02BC01	9 (3.6)	15 (4.2)
Pantoprazole	A02B	1(0.4)	1 (0.3)
Paracetamol	N02BE01	1 (0.4)	12 (3.4)
Propranolol	C07A	3 (1.2)	3 (0.8)
Ranitidine	A02BA02	3 (1.2)	3 (0.8)
Salbutamol	R03	1(0.4)	3 (0.8)
Scopolamine	A03BB01	3 (1.2)	6 (1.7)
Sertraline	N06A	2 (0.8)	1 (0.3)
Simvastatin	C10AA01	9 (3.6)	4 (1.1)
Zolpidem	N05	5 (2.0)	7 (2.0)
Others		53 (21.0)	78 (21.8)
Total		251 (100)*∗∗*	358 (100)*∗∗*

^**∗**^Classification according to the Anatomical Therapeutic Chemical Code (ATC code) [24].

^*∗∗*^Without statistical meaningful difference between the amount of medications used in the groups (student t; p = 0.4470).

**Table 4:** Herbal medicines reported by the elderly participants on the structured questionnaire (N=123), Macapá, Brazil, 2016-2017.

Herbal medicines*∗*	popular name	Therapeutic Indications	Structured questionnaire	
			N	%
*Lippia alba *(Mill.) N.E. Br	Cidreira	Relaxation and digestive problems	63	19.9
*Peumus boldus *Molina	Boldo	Digestive and liver problems	35	11.1
*Cymbopogon citratus *(DC.) Stapf	Capim-marinho	Relaxation and digestive problems	31	9.8
*Carapa guianensis *Aubl.	Andiroba	Inflammation, bruises	18	5.7
*Matricaria chamomilla* L.	Camomila	Relaxation, nausea, colic	11	3.5
*Stryphnodendron adstringens *(Mart.)	Barbatimão	Infections, wound healing, pain, inflammation	11	3.5
*Copaifera langsdorffii* Desf.	Copaíba	Inflammation, Infections	9	2.8
*Cinnamomum zeylanicum* Blume	Canela	Digestive, energy/ stimulation problems	8	2.5
*Arrabidaea chica *(Bonpl.) Verl.	Pariri	Pain, fever, inflammation and/or spams	8	2.5
*Dysphania anthelmintica* (L.) Mosyakin & Clemants	Mastruz	Parasitic infection	7	2.2
*Costus spicatus* (Jacq.) Sw.	Cana-do-brejo	Kidney problems (diuretic effect)	6	1.9
*Veronica officinalis* L.	Verônica	Pain, fever, inflammation	6	1.9
*Mentha alaica* Boriss.	Hortelã	Nause, digestive problems	6	1.9
*Phyllanthus niruri L.*	Quebra-Pedra	Kidney problems (diuretic and stone-preventing effects)	5	1.6
*Aesculus hippocastanum* L.	Castanha da Índia	Blood circulation/varicose, inflammation	4	1.3
*Pentaclethra eetveldeana* De Wild. & T. Durand	Pracaxi	Infections	4	1.3
*Zingiber officinale* Roscoe	Gengibre	Energy/stimulation problems	2	0.6
*Aloe vera* (L.) Burm.f.	Babosa	Healing, protector of the gastric and intestinal mucosa	2	0.6
Others			68	21.5
Total			316	100

^*∗*^The classification of botanical names was according to THE PLANTS LIST® database [25]. The botanical identification of the herbal medicines obtained in pharmacies was derived from the labels/packages, and the herbal medicines obtained in gardens, fairs, and popular markets were identified by visual stimuli, in the form of pictures and images from online herbariums (reportedly used by the interviewees to provide relief against illnesses).

**Table 5:** Characteristics of herbal medicine use reported by the elderly participants on the structured questionnaire. Macapá, Brazil, 2016-2017.

**Characteristics**	**Structured questionnaire **	
	**N**	%
**Origin of herbal medicines** **∗**		
Fairs or popular markets	95	51.6
Garden	68	37.0
Drugstore	21	11.4
Total	184	100
**Presentations** **∗** **∗**		
Infusion/Tea	188	59.5
Plant extracts	87	27.5
Gel with plant ingredients	23	7.3
Oils	18	5.7
Total	316	100
**Mode of administration** **∗** **∗**		
Oral	266	84.2
Topic	50	15.8
Total	316	100

*∗*Some herbal medicines, according to the self-report of the elderly, were obtained in more than one place according to availability.

*∗∗*The 316 herbal medicines used by the elderly were classified according to the mode of preparation (pharmaceutical form) and the route of administration according to the structured questionnaire.

**Table 6:** Medications used in combination or not with herbal medicines by elderly participants as determined by pharmacotherapeutic follow-up (N=38), Macapá, Brazil, 2016-2017.

**Medications**	**ATC** **∗**	**Pharmacotherapeutic follow-up**	
		**Only medication use ** **n (**%**)**	**Herbal medicines and medication use n (**%**)**
Losartan	C09AA01	3 (11.5)	25 (12.3)
Omeprazole	A02BC01	4 (15.4)	24 (11.8)
Diclofenac	M01AB05	2 (7.7)	15 (7.3)
Glibenclamide	A10BB01	3 (11.5)	12 (5.9)
Hydrochlorothiazide	C03AA03	2 (7.7)	12 (5.9)
Insulin	A10AC01	1 (3.9)	9 (4.4)
Acetylsalicylic acid	N02BA01	3 (11.5)	9 (4.4)
Nimesulide	M01AX17	1 (3.9)	7 (3.4)
Others		7 (26.9)	91 (44.6)
Total		26 (100)*∗∗*	204 (100)*∗∗*

*∗*Classification according to the Anatomical Therapeutic Chemical Code (ATC code) [24].

*∗∗*There was statistical meaningful difference between the amount of medications used in the groups (Student t; p = 0.0004).

**Table 7:** Herbal medicines most frequently used by elderly participants as determined through pharmacotherapeutic follow-up (N=33), Macapá, Brazil, 2016-2017.

**Herbal medicines**	**Popular name**	**Indications**	**Pharmacotherapeutic** **follow-up**	
			**N**	%
*Peumus boldus* Molina	Boldo	Digestive and liver problems	14	19.4
*Lippia alba *(Mill.) N.E. Br	Cidreira	Relaxation and digestive problems	12	16.7
*Cymbopogon citratus *(DC.) Stapf	Capim-marinho	Relaxation and digestive problems	12	16.7
*Carapa guianensis *Aubl.	Andiroba	Inflammation, bruises	11	15.3
*Phyllanthus niruri *L.	Quebra-Pedra	Kidney problems (diuretic and stone-preventing effects)	6	8.3
*Matricaria chamomilla* L.	Camomila	Relaxation, nausea, colic	3	4.2
Others			14	19.4
Total			72	100

^*∗*^The classification of botanical names was according to THE PLANTS LIST® database [25]. The botanical identification of the herbal medicines obtained in pharmacies was derived from the labels/packages, and the herbal medicines obtained in gardens, fairs, and popular markets were identified by visual stimuli, in the form of pictures and images from online herbariums (reportedly used by the interviewees to provide relief against illnesses).

**Table 8:** Frequency of ADRs in elderly participants based on the ADR causality assessment methods WHO [19, 20], Macapá, Brazil, 2016-2017.

**ADR causality assessment**	**Structured questionnaire **		**Pharmacotherapeutic ** **follow-up **	
	**Only medication use n (**%**)**	**Herbal medicines and medication** **use n (**%**)**	**Only medication use n (**%**)**	**Herbal medicines and medication** **use n(**%**)**
Defined	0 (0.0)	0 (0.0)	2 (50.0)	9 (39.1)
Probable	4 (13.8)	2 (3.5)	1 (25.0)	8 (34.8)
Possible	21 (72.4)	46 (80.7)	1 (25.0)	5 (21.3)
Unlikely	4 (13.8)	9 (15.8)	0 (0.0)	1 (4.3)
**Total**	**29 (100)**	**57 (100)**	**4 (100)**	**23 (100)**

**Table 9:** Frequency of ADRs confirmed/defined in elderly participants based on the terminology for coding clinical information in relation to medical therapy [26], Macapá, Brazil, 2016-2017.

**Variable**	**Structured questionnaire n (**%**)**	**Pharmacotherapeutic follow-up n (**%**)**
Nervous system	28 (38.4)	5 (22.7)
Digestive system	19 (26.0)	8 (36.4)
Symptoms, signs and abnormal clinical and laboratory findings, not classified elsewhere	13 (17.8)	5 (22.7)
Circulatory system	7 (9.6)	2 (9.1)
Skin and subcutaneous tissue	5 (6.8)	2 (9.1)
Respiratory system	1 (1.4)	0 (0.0)
**Total**	**73 (100)**	**22 (100)**

## Data Availability

The data used to support the findings of this study are available from the corresponding author upon request.

## References

[1] WHO Traditional medicine. http://www.who.int/mediacentre/.

[2] Oliveira E. C. S., Trovão D. M. B. M. (2009). O uso de plantas em rituais de rezas e benzeduras: um olhar sobre esta prática no estado da Paraíba. *Revista Brasileira de Biociências*.

[3] Da Silva G. S. (2010). Benzedores e raizeiros: saberes partilhados na comunidade remanescente de quilombo de Santana da Caatinga. *Revista Mosaico*.

[4] Zank S., Hanazaki N., Bussmann R. (2017). The coexistence of traditional medicine and biomedicine: A study with local health experts in two Brazilian regions. *PLoS ONE*.

[5] Mignone J., Bartlett J., O'Neil J., Orchard T. (2007). Best practices in intercultural health: Five case studies in Latin America. *Journal of Ethnobiology and Ethnomedicine*.

[6] Vandebroek I. (2013). Intercultural health and ethnobotany: How to improve healthcare for underserved and minority communities?. *Journal of Ethnopharmacology*.

[7] da Rosa C. Traditional Medicine and Complementary/Alternative Medicine in Primary Health Care: The Brazilian Experience. http://www.intechopen.com/books/primary-care-at-aglance-hot-topics-and-new-insights/traditional-medicine-and-complementary-alternative-in-primary-healthcare-the-brazilian-experience.

[8] World Health Organization (WHO) Traditional Medicine Strategy 2002–2005, Geneva, Switzerland. http://whqlibdoc.who.int/hq/2002/who_edm_trm_2002.1.pdf.

[9] World Health Organization (2004). *WHO Guidelines on Safety Monitoring of Herbal Medicines in Pharmacovigilance Systems*.

[10] World Health Organization National Policy on Traditional Medicine And Regulation of Herbal Medicines. http://apps.who.int/medicinedocs/pdf/s7916e/s7916e.pdf.

[11] Ministério da Saúde Portaria n° 971, de 3 de maio de 2006. Aprova a Política Nacional de Práticas Integrativas e Complementares (PNPIC) no Sistema Único de Saúde. Brasília (DF). http://bvsms.saude.gov.br/bvs/saudelegis/gm/2006/prt0971_03_05_2006.html.

[12] Ministério da Saúde (2010). *Decreto n^º^ 5.813, de 22 de junho de 2006. Aprova a Política Nacional de Plantas Medicinais e Fitoterápicos e dá outras providências*.

[13] Rodrigues E., Barnes J. (2013). Pharmacovigilance of herbal medicines: The potential contributions of ethnobotanical and ethnopharmacological studies. *Drug Safety*.

[14] Lanini J., Duarte-Almeida J. M., Nappo S., Carlini E. A. (2009). “Natural and therefore free of risks” - Adverse effects, poisonings and other problems related to medicinal herbs by “ raizeiros” in Diadema/SP". *Revista Brasileira de Farmacognosia*.

[15] Barnes J. (2003). Pharmacovigilance of herbal medicines: A UK perspective. *Drug Safety*.

[16] Mangoni A. A., Jackson S. H. D. (2004). Age-related changes in pharmacokinetics and pharmacodynamics: basic principles and practical applications. *British Journal of Clinical Pharmacology*.

[17] Davies E. A., O'Mahony M. S. (2015). Adverse drug reactions in special populations - The elderly. *British Journal of Clinical Pharmacology*.

[18] van den Akker M., Buntinx F., Knottnerus J. A. (1996). Comorbidity or multimorbidity. *The European Journal of General Practice*.

[19] Edwards I. R., Aronson J. K. (2000). Adverse drug reactions: definitions, diagnosis, and management. *The Lancet*.

[20] World Alliance for Patien Safety (2005). *WHO draft guidelines for adverse event reporting and learning systems, From information to action*.

[21] Organização Pan-Americana da Saúde (2011). Boas práticas de farmacovigilância para as Américas. *Rede PAHRF Documento Técnico Nº 5*.

[22] Banco Central do Brasil Câmbio e capitais internacionais. Taxas de câmbio. http://www.bcb.gov.br.

[23] Kennerfalk A., Ruigómez A., Wallander M.-A., Wilhelmsen L., Johansson S. (2002). Geriatric drug therapy and healthcare utilization in the United Kingdom. *Annals of Pharmacotherapy*.

[24] WHO Collaboranting Centre for Drug Statistics Methodology ATC codes 2003. http://www.whocc.nmd.no.

[25] The Plants List®Database The Plant List is a working list of all known plant species. http://www.theplantlist.org.

[26] World Health Organization International monitoring of adverse reactions to drugs: adverse reaction terminology.

[27] Instituto Brasileiro de Geografia e Estatística Análise do panorama das cidades brasileiras. Dados populacionais de Macapá. https://ibge.gov.br./.

[28] Shaw D. (2010). Toxicological risks of Chinese herbs. *Planta Medica*.

[29] Mulkalwar S., Munjal N., Worlikar P., Behera L. (2013). Pharmacovigilance in India. *Medical Journal of Dr. D.Y. Patil University*.

[30] (2016). *Rdc 98/2016 Resolução Da Diretoria Colegiada – Rdc N° 98, De 1° De Agosto De 2016*.

[31] Cameron S., Turtle-Song I. (2002). Learning to write case notes using the SOAP format. *Journal of Counseling & Development*.

[32] Machuca M., Fernández-Llim F., Faus M. J. (2003). *Método Dáder. Guía de seguimiento fármacoterapéutico*.

[33] Strand L. M., Cipolle R. J., Morley P. C., Frakes M. J. (2004). The impact of pharmaceutical care practice on the practitioner and the patient in the ambulatory practice setting: Twenty-five years of experience. *Current Pharmaceutical Design*.

[34] Cipolle R. J., Strand L L., Morley P. (2014). *Pharmaceutical Care Practice: The Clinican’S Guide*.

[35] Riba R. F., Estela A. C., Esteban M. L. S. (2000). Intervenciones farmacéuticas (parte I): metodología y evaluación. *Farmacia Hospitalaria*.

[36] Sabater D., Fernandez-LLimos F., Parras M., Faus M. J. (2005). Types of pharmacist intervention in pharmacotherapy follow-up. *Seguimiento Farmacoteraéutico*.

[37] Ministério da Saúde (2010). *Portaria nº. 886, de 20 de abril de 2010. Institui a Farmácia Viva no âmbito do Sistema Único de Saúde (SUS)*.

[38] Agência Nacional de Vigilância Sanitária [Brasil] (2000). *Esolução Da Diretoria Colegiada Nº18, De 03 De Abril De 2013. Dispõe Sobre as Boas Práticas De Processamento E Armazenamento De Plantas Medicinais, Preparação E Dispensação De Produtos Magistrais E Oficinais De Plantas Medicinais E Fitoterápicos Em Farmácias Vivas No Âmbito Do Sistema Único De Saúde (SUS)*.

[39] Agência Nacional de Vigilância Sanitária (ANVISA) Gerenciamento do Risco em Farmacovigilância. http://portal.anvisa.gov.br.

[40] Ratajczak H. V. (2004). Drug-induced hypersensitivity: Role in drug development. *Toxicological Reviews*.

[41] Resende L. S., Santos-Neto E. T. (2015). Risk factors associated with adverse reactions to antituberculosis drugs. *Jornal Brasileiro de Pneumologia*.

[42] Umair M., Altaf M., Abbasi A. M., Bussmann R. (2017). An ethnobotanical survey of indigenous medicinal plants in Hafizabad district, Punjab-Pakistan. *PLoS ONE*.

[43] Doyal L., Kickbusch I., Kari A. (2015). Understanding gender, health, and globalization: opportunities and challenges. *Globalization, Women, and Health in the 21st Century*.

[44] Abdelhalim A., Aburjai T., Hanrahan J., Abdel-Halim H. (2017). Medicinal plants used by traditional healers in Jordan, the Tafila region. *Pharmacognosy Magazine*.

[45] Delgoda R., Younger N., Barrett C., Braithwaite J., Davis D. (2010). The prevalence of herbs use in conjunction with conventional medicines in Jamaica. *Complementary Therapies in Medicine*.

[46] Picking D., Younger N., Mitchell S., Delgoda R. (2011). The prevalence of herbal medicine home use and concomitant use with pharmaceutical medicines in Jamaica. *Journal of Ethnopharmacology*.

[47] Bruno J. J., Ellis J. J. (2005). Herbal use among US elderly: 2002 National Health Interview Survey. *Annals of Pharmacotherapy*.

[48] de Medeiros P. M., Ladio A. H., Albuquerque U. P. (2013). Patterns of medicinal plant use by inhabitants of Brazilian urban and rural areas: a macroscale investigation based on available literature. *Journal of Ethnopharmacology*.

[49] Di Stasi L. C., Oliveira G. P., Carvalhaes M. A. (2002). Medicinal plants popularly used in the Brazilian Tropical Atlantic Forest. *Fitoterapia*.

[50] Olisa N. S., Oyelola F. T. (2009). Evaluation of use of herbal medicines among ambulatory hypertensive patients attending a secondary health care facility in Nigeria. *International Journal of Pharmacy Practice*.

[51] Kassaye K. D., Amberbir A., Getachew B., Mussema Y. (2006). A historical overview of traditional medicine practices and policy in Ethiopia. *Ethiopian Journal of Health Development*.

[52] Carvalho Mastroianni P. D., Rossi Varallo F., Amaral Costa M., Da Silva Sacramento L. V. (2017). Development of Instrument to Report And Assess Causality of Adverse Events Related to Herbal Medicines. *Revista Vitae*.

[53] Naranjo C. A., Busto U., Sellers E. M. (1981). A method for estimating the probability of adverse drug reactions. *Clinical Pharmacology & Therapeutics*.

[54] Karch F. E., Lasagna L. (1976). Evaluating Adverse Drug Reactions. *Adverse Drug Reaction Bulletin*.

[55] Kosalec I., Cvek J., Tomic S. (2009). Contaminants of medicinal herbs and herbal products. *Archives of Industrial Hygiene and Toxicology*.

[56] ICH Topic E2E pharmacovigilance planning (PVP).

